# Experimental Study on the Fatigue Crack Propagation Rate of 925A Steel for a Ship Rudder System

**DOI:** 10.3390/ma17081808

**Published:** 2024-04-15

**Authors:** Li Yu, Wenyong Guo, Chenghao Cao, Min Li, Zhe Wu, Te Wang, Hantao Chen, Xinglong Pan

**Affiliations:** 1 College of Power Engineering, Naval University of Engineering, Wuhan 430030, China; 0908042009@nue.edu.cn (L.Y.); guowy202@163.com (W.G.); 1920191197@nue.edu.cn (C.C.); 13876768712@163.com (M.L.); 1920191013@nue.edu.cn (Z.W.); 2 Hubei Zerong Testing Technology Co., Ltd., Xiangyang 441001, China; wt1259482549@163.com

**Keywords:** steels for rudders, 925A steel, Arctic, low-temperature fatigue crack propagation, crack propagation rate

## Abstract

The low-temperature fatigue crack propagation rate of 925A steel, as a rudder steel for polar special ships, has a crucial impact on the evaluation of the fatigue strength of polar ships. The purpose of this article is to study the fatigue crack propagation rate of 925A steel under different low-temperature conditions from room temperature (RT) to −60 °C. The material was subjected to fatigue crack propagation tests and stress intensity factor tests. The experimental tests were conducted according to the Chinese Standard of GB/T6398-2017. The results show that as the temperature decreases, the lifespan of 925A increases. Within a certain stress intensity factor, as the temperature decreases, the fatigue crack propagation rate decreases. At −60 °C, it exhibits ductile fracture; within normal polar temperatures, it can be determined that 925A meets the requirements for low-temperature fatigue crack propagation rates in polar regions. However, in some extreme polar temperatures below −60 °C, preventing brittle failure becomes a key focus of fatigue design. Finally, the fatigue crack propagation behavior at the microscale of 925A steel at low temperatures was described using fracture morphology. The experimental data can provide reference for the design of polar ships to further resist low-temperature fatigue and cold brittle fracture.

## 1. Introduction

With the advancement of global climate change and the increase in polar exploration activities, the characteristics of Arctic shipping routes have become increasingly prominent, and an increasing number of countries are actively striving to develop Arctic shipping routes [1]. The complex environment beneath the Arctic ice sheet makes it difficult to detect submarines, making them highly valuable for strategic planning. Submarines from the UK, US, and Russia have been operating under the thick ice layer on the Arctic Ocean for years, and nuclear submarine strategists regard this area as an important hiding place and attack site. Although nuclear submarines can remain underwater for a long time without floating, they must float to receive base information and to launch missiles. The rudder is an important mechanical system used to control the course and attitude of ships. This system consists of multiple components such as a servo, transmission shaft, bearing sleeve, and push rod, which need to withstand complex alternating loads during operation [2]. The elevator is a balanced non-watertight rudder, located entirely at the stern of the ship. The double rudder blades form a trapezoidal structure and is connected by shafts fixed on the horizontal stabilizing wings on both sides of the stern. The stern elevator system is used to overcome the torque on the rudder stock for steering. Among the components that comprise the rudder mechanism, the guide rod plays an important role in transmitting the torque of the rudder and is a key component of the transmission process in the rudder system. Breaking and floating ice have both military and scientific significance. Submarines collect a large amount of sea ice data during their ascent, making these vessels valuable resources for climate change researchers. In low-temperature polar environments, these components face severe challenges, such as decreased material properties, increased brittleness, and fatigue crack propagation. Studying the low-temperature fatigue performance of these materials is highly important.

Extreme conditions, such as low temperature, high pressure, and high corrosion, in polar environments, have a significant impact on the fatigue life of rudder materials for ships [3]. Thus, 925A steel has become an important material for ship components, such as rudder transmission devices, rudder blades, and rudder stocks, due to its outstanding mechanical properties, which may collide with sea ice during navigation and cause fatigue problems [4]. To improve the safety and reliability of ships in polar environments, this article aims to study the low-temperature fatigue performance of ship rudder materials in polar low-temperature environments, provide low-temperature fatigue parameters for ship polar navigation design, analyze their influencing factors and mechanisms through experimental methods, and further clarify the fatigue performance of rudder materials under low-temperature conditions. Further, this article provide a reference for te low-temperature fatigue testing of future rudder stock mechanisms.

Fatigue fracture is one of the main modes of fracture in metal components, and the massive majority of the damage is caused by fatigue. The fatigue crack propagation rate, as an indicator of the fracture mechanical performance of a material, is very important for accurately evaluating the service life of a component. According to the ABS (American Bureau of Shipping) regulations for the design of ships in low-temperature polar regions, the minimum temperature considered in the process is −60 °C [5]. Therefore, it is particularly important to study the fatigue strength of 925A steel used in ship rudder systems at temperatures of at least −60 °C. The experimental tests in the article were conducted according to the Chinese Standard of GB/T6398-2017 [6].

From the perspective of studying materials based on fracture mechanics, global scholars have studied the fatigue crack propagation performance of different steels under room-temperature and low-temperature conditions.

Galatolo [7] critically analyzed the theoretical research in the literature, transformed it into analytical models, and conducted fatigue tests to validate these models. And use the model to develop the PISA code for simulating crack propagation in aerospace metal components. Cavuoto’s [8] research has shown that the determination of crack trajectories is important for the design, accounting for the failure of materials and mechanical components. Seifi [9] conducted fatigue tests on the fatigue crack propagation of hollow prefabricated notched plates with multi-point damage to evaluate the influence of some parameters. The numerical results were verified through experiments. Anasori [10] studied the fatigue crack propagation of 2198-T8 aluminum–lithium alloy riveted lap joints through fracture analysis. The study also compared the fatigue behavior of AA2198-T8 and AA2024-T3 alloys, emphasizing the differences in fracture surface morphology, crack propagation rate, and the presence of micro and interlayer cracks. Alqahtani [11] conducted a comprehensive study on the effects of temperature and humidity on Al6082 alloy FCGR. An empirical model was also established to accurately predict the fatigue life cycle values under these environmental conditions.

Song W. et al. [12] examined the fatigue crack growth behaviors of different zones in an overmatched welded joint composed of D32 marine structural steel. These scholars provided valuable data for the safe design and life prediction of welded marine structures. Braun M. et al. [13] employed 26 series of fatigue tests on welded and non0welded specimens containing notches located in different material zones. Guo Y. et al. [14] conducted axial loading fatigue tests with different stress ratios. A reproducible analysis approach for predicting the fatigue lives of welded joints was introduced. The researchers investigated the fatigue behavior and predicted the lifespan of carburized Cr-Ni gear steel in the VHCF regime. Lv C. et al. [15] investigated fatigue crack propagation analysis models and fatigue crack propagation simulations based on the XFEM. The consistency between the finite element simulation and experimental data was verified through a comparison with the experimental data. Hu et al. [16] provided a comprehensive review of fatigue experiments conducted on high-entropy alloys over the past two decades. The focuses of this paper include crack initiation behaviors, crack propagation modes, and fatigue life prediction models.

Zong L. et al. studied the fatigue crack propagation performance characteristics of the high-strength structural steels Q690D [17], WNQ570 [18], and Q345qD [19] through fatigue crack propagation rate experiments and obtained the Paris parameters *m* and *C* of the materials; scholars have determined the fatigue crack propagation rate parameters of specimens with different thicknesses and stress ratios. Compared with several other commonly used structural steels, it is believed that Q690D has better resistance to fatigue crack propagation. Wang Y. et al. [20] summarized the research results of global scholars on the low-temperature fatigue performance of building and bridge steel structures, indicating that the low-temperature impacted the fatigue performance of steel structures, thus clarifying the future research direction of this topic. Liao X. et al. [21] studied the fatigue crack propagation behavior of Q345qD bridge steel in low-temperature environments. Through fatigue crack propagation rate tests between approximately room temperature and −60 °C, it was determined that the steel had good resistance to low-temperature fatigue crack propagation, providing a reference for the design of methods for preventing fatigue and cold brittle fracture at low temperatures for materials used in polar environments.

Tong L. et al. [22] analyzed the fatigue performance of domestic high-strength structural steel through fatigue crack propagation rate tests and used different methods for data processing to compare and analyze their advantages and disadvantages in terms of fatigue performance. Fan Y. et al. [23] studied the corrosion fatigue crack propagation behaviors of 304, 316, and 321 stainless steels, showing that the corrosion fatigue crack propagation rates of the three types of stainless steels in seawater environments were significantly greater than those in air; furthermore, the corrosion fatigue crack propagation rates of the materials complied with the Paris Law. Zhong Y. et al. [24] conducted fatigue crack propagation tests on EH36 steel under different stress ratios in three marine corrosion environments: an air environment, a seawater immersion environment, and a seawater splash environment. The scholars observed that the fatigue life of EH36 steel increased with the increasing stress ratio in all three environments, but the effect of corrosion on the fatigue performance gradually increased. Liu J. et al. [25] used Ansys Workbench and n Code Design Life software 2021R1 version to study the fatigue life of defective welds in transformer oil tanks and concluded that the vibration peak value and defect size significantly impacted the fatigue life of oil tank welds.

Chen B. et al. [26] used n Code software to analyze the fatigue life of defective fillet welds in transformer oil tanks and concluded that the greater the difference between the elastic modulus of the slag inclusion and the weld seam, the more likely it was to cause fatigue failure. Xu L. et al. [27] validated incremental plasticity by comparing the crack propagation rate curve of a 7N01 aluminum alloy with that of linear elastic mechanics through computer simulation. The rationality of using the Δ*J*-integral as a driving force parameter for crack propagation provided a research basis for further exploring the mechanism of crack propagation. Bai L. et al. [28] conducted a fatigue crack propagation rate analysis on welded structures, revealing the influences of various factors, such as the welding plate thickness, reinforcement height, and stress ratio, on the fatigue crack propagation rate. Wang K. et al. [29] conducted fatigue crack propagation rate tests on titanium alloys at room temperature and low temperatures, finding that titanium alloys met the requirements for a low-temperature fatigue crack propagation rate in polar regions and providing a reference for the design of polar icebreakers to resist low-temperature fatigue and cold brittle fracture. However, under low-temperature conditions, there is still a lack of systematic and comprehensive research, clear conclusions, and reliable data on the fatigue crack propagation performance of 925A steel used in ship rudder systems.

15CrNi3MoV steel is also known as 925A steel. This material is the most critical structural material in modern ship construction. The performance of this steel directly affects the improvement in the tactical performance of the ship. This steel does not undergo brittle failure and has very strict toughness requirements. As a material for ship structures, ship structural steel must have sufficient strength and toughness, good processability, and resistance to seawater corrosion. In the investigation, 925A steel was used as the material for ship rudder stock. 925A steel has the characteristics of high strength, good toughness, resistance to seawater corrosion, and excellent comprehensive performance. Therefore, 925A steel was taken as the research object to complete fatigue crack propagation rate tests at three temperatures: 20 °C, −20 °C, and −60 °C. The influence of low temperature on the fatigue crack propagation behavior of 925A steel welding materials was explored, providing a research basis for further studying the mechanism of rudder stock fatigue.

## 2. Materials and Test Methods

### 2.1. Test Materials

This experiment used 925A steel (certified by Wuchang shipbuilding indusday (CSSC), Wuhan, China) with a plate thickness of 25 mm. The chemical composition parameters of 925A are shown in Table 1.

To obtain the basic mechanical properties of 925A steel, including yield strength, tensile strength, plane strain fracture toughness, and fatigue crack propagation rate, conventional room temperature static load tensile and impact tests were first conducted on 925A steel; these tests were followed by fracture toughness tests.

### 2.2. Fatigue Crack Propagation Rate Experiment

**Figure 1:**
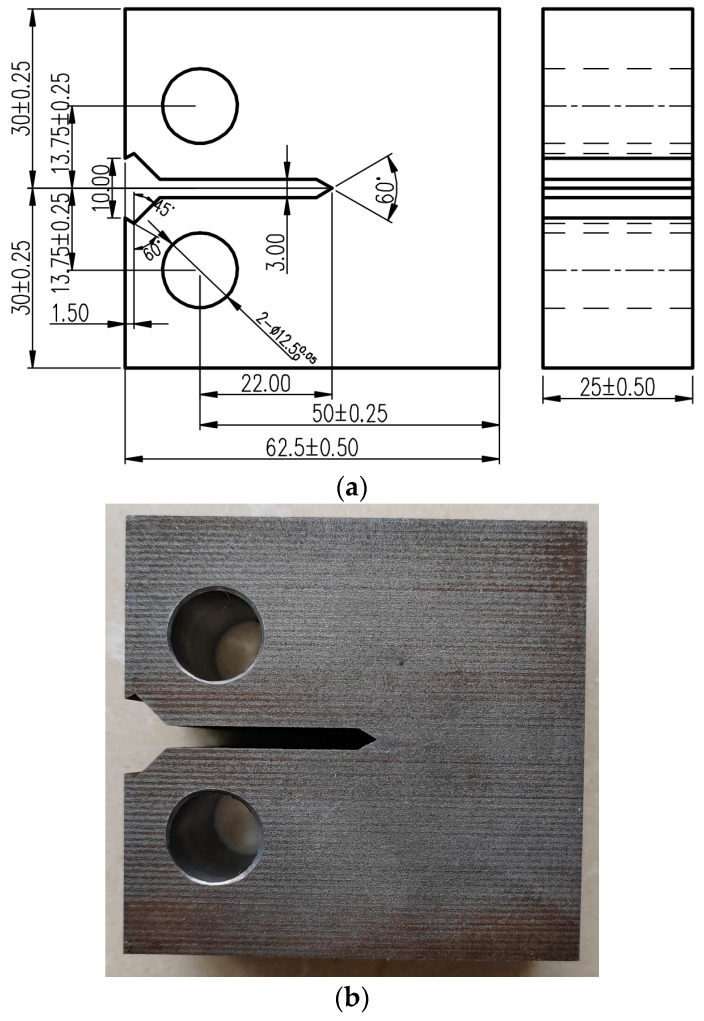
The geometry of the C(T) specimen: (**a**) sample size diagram (unit mm); (**b**) sample physical image (1:1 scale).

The fatigue crack propagation test was conducted following the standard test method for the fatigue crack propagation rate of metal materials (GB/T6398-2017) [6]. A standard compact tensile C(T) specimen was used on 25-mm-thick 925A steel. The specific dimensions of the specimen are shown in Figure 1. The crack propagation direction of the specimen was perpendicular to the rolling direction, and the prefabricated fatigue crack length was approximately 2~3 mm. According to the research results of Wang K. [30] and Mendes P. [31], at different temperatures, the fatigue crack propagation rates with different stress ratios had small differences, and a large stress ratio could reduce the closure effect. Therefore, a stress ratio of 0.1 was selected for the low-temperature crack propagation rate test of 925A steel. The test samples were tested at three temperatures—room temperature (20 °C), −20 °C, and −60 °C—with three samples at each temperature for a total of 9 effective samples.

**Figure 2:**
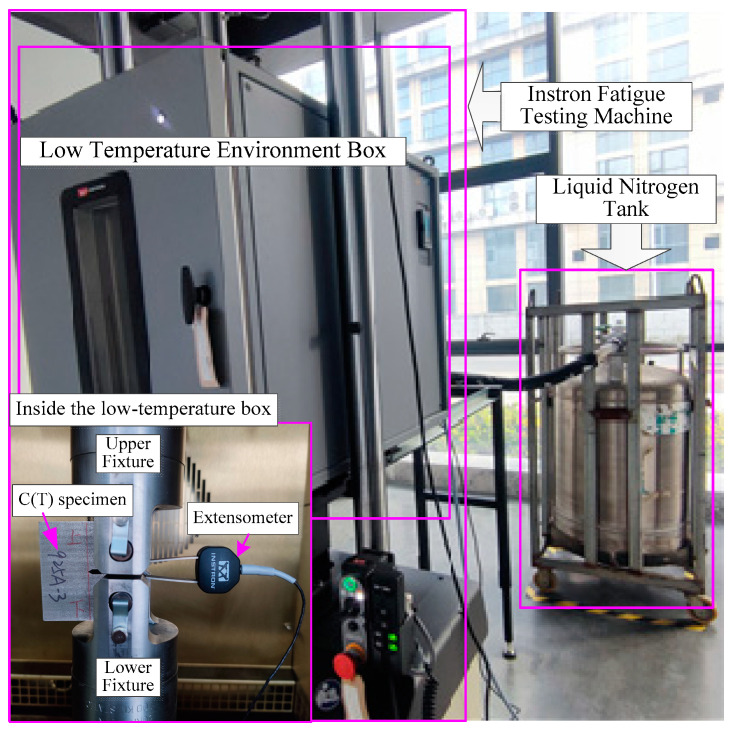
Fatigue crack growth test at low temperatures.

The experiment was completed on an Instron electrohydraulic servo fatigue testing machine, with a loading frequency of 10 Hz and an extensometer range of 4 mm, all of which met the test requirements, as shown in Figure 2. In the experiment, a mixture of liquid nitrogen and air was used to simulate a low-temperature environment, and the temperature was controlled through a low-temperature environment box. An extensometer was used to measure the crack propagation length based on the principle of flexibility. After the experiment, the pre-fatigue crack length and propagation termination crack length of the C(T) specimen fracture were measured using a tool microscope. Then, curvature correction was performed. According to GB/T6398-2017 [6], the seven-point incremental term fitting method was used to fit the curvature-corrected *a–N* curve, and the fatigue crack propagation rate *da*/*dN* and corresponding stress intensity factor amplitude Δ*K* were calculated.

## 3. Results and Discussion

### 3.1. Tensile and Impact Test

The results of the basic mechanical performance tests, including the static tensile test and impact test, are shown in Table 2. It provides effective data for subsequent fatigue crack propagation rate experimental research on 925A steel.

From the test data, the strength of 925A steel increases with the decrease in temperature, especially when it reaches −60 °C, and the tensile strength increases significantly. However, no obvious yield phenomenon was found during the test, so the conditional yield strength value (σ_0.2_) was used as the yield limit of the material. As the temperature gradient decreases, its impact performance also decreases. The elastic modulus also shows a similar change. The Poisson’s ratio did not show significant changes, but since the specimens used in this experiment were plate-shaped specimens, there was a slight difference between the test values of the column-shaped specimens, but it remained around 0.3.

### 3.2. Fatigue Crack Propagation Rate Test

**Figure 3:**
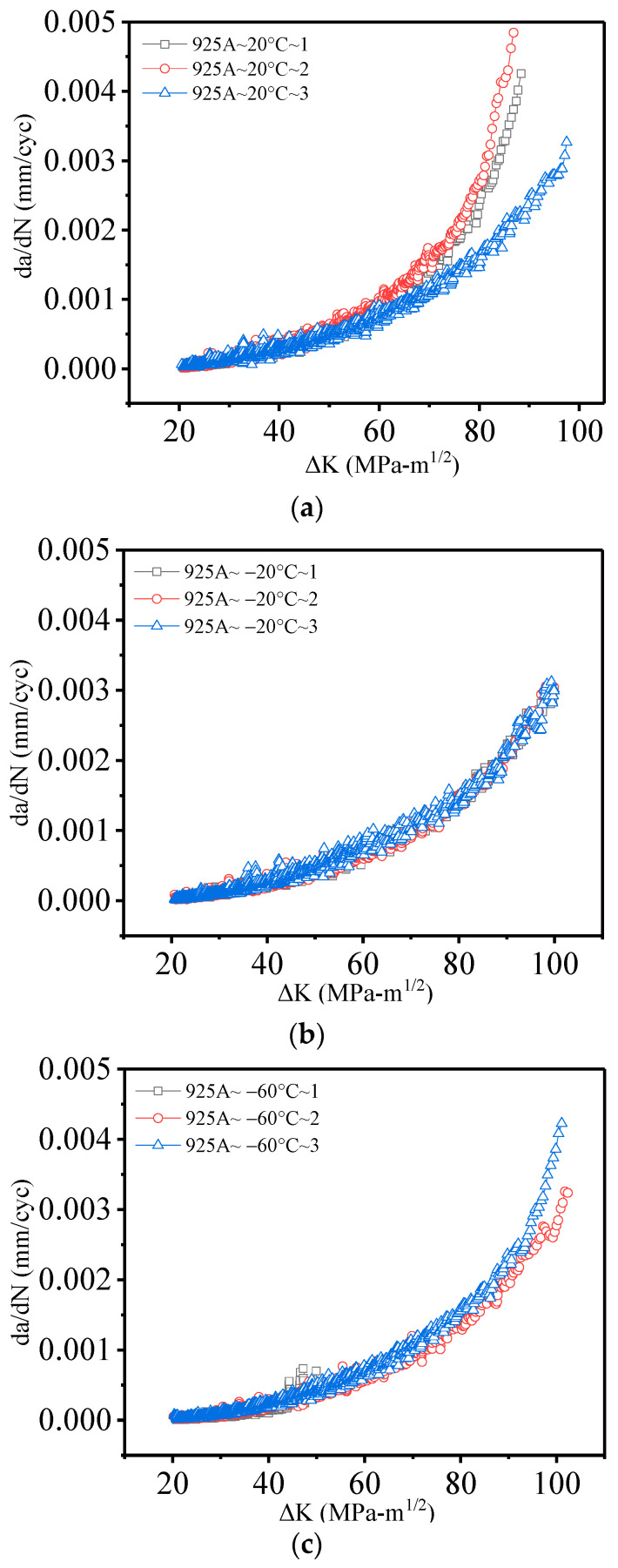
Crack propagation results of 925A steel: (**a**) 20 °C; (**b**) −20 °C; (**c**) −60 °C.

Based on a series of data processing methods, such as the seven-point incremental equation, the fatigue propagation *a–N* curves of 925A steel C(T) specimens under different temperature conditions with a stress ratio of R = 0.1 have been obtained. In addition, the crack propagation results of 925A steel have been recorded, as shown in Figure 3. As the temperature decreases, the lifespan of the 925A steel increases, with the maximum lifespan occurring at −60 °C. However, it is difficult to determine the effect of low temperature on the fatigue crack propagation rate solely from the perspective of the *a–N* curve. The fatigue crack propagation rate curve is needed to determine the impact of low temperature on fatigue performance.

### 3.3. Effect of Low Temperature on Fatigue Crack Propagation Behavior

It has been noted [32,33,34,35,36] that low-alloy structural steel has a fatigue brittle–ductile transition temperature. Specifically, when the ambient temperature is higher than the brittle–ductile transition temperature, the fatigue crack propagation rate decreases with decreasing temperature. In addition, when the ambient temperature is lower than the brittle–ductile transition temperature, the fatigue crack propagation rate increases with decreasing temperature. From the *a–N* curve in Figure 3, the slope of the curve (*da*/*dN*)*_i_* is calculated using the line-cut method, and the value of the crack propagation rate is obtained. The value of the stress intensity factor range (Δ*K*)*_i_* is calculated using Equation (1), and the *a–N* curve is processed using the line-cut method to construct a double logarithmic curve of the fatigue crack propagation rate *da*/*dN* for 925A steel.

(1) $$\frac{da}{dN}=C(\Delta K{)}^{m},$$

where Δ*K* represents the range of variation in the stress intensity factor at the crack tip, and *C* and *m* are constants related to the material.

According to the formula, the curve of the crack propagation rate is drawn, the logarithms of its two sides are taken, and the linear Equation (2) is obtained:

(2) log(da/dN)=logC+mlog(ΔK).

For the C(T) specimen, Equation (3) can be used to calculate Δ*K*:

(3) $$\Delta K=\frac{\Delta P}{BW^{1/2}}g\left(\frac{a}{W}\right),$$

where $g\left(\frac{a}{W}\right)$ indicates the shape factor of the standard specimen. The formula for calculating the shape factor of the C(T) samples is as follows [37]:

$$g\left(\frac{a}{W}\right)=\frac{\left(2+a\right)\left(0.886+4.64a-13.32a^{2}+14.72a^{3}-5.6a^{4}\right)}{{\left(1-a\right)}^{3/2}}.$$

Equation (3) is an equation used to calculate the range of stress intensity factors (∆*K*), where *a* = *a*/*W* represents the ratio of the crack size to the specimen width, Δ*P* represents the range of the applied fatigue cyclic load, *B* represents the thickness of the specimen, and *W* represents the width of the specimen. Finally, for the *da/dN −* Δ*K* curve, linear fitting is performed based on the Paris law in a logarithmic coordinate system [37]. The purpose of this equation is to calculate the relationship between the range of stress intensity factors and the crack size, specimen geometry, and applied load.

**Figure 4:**
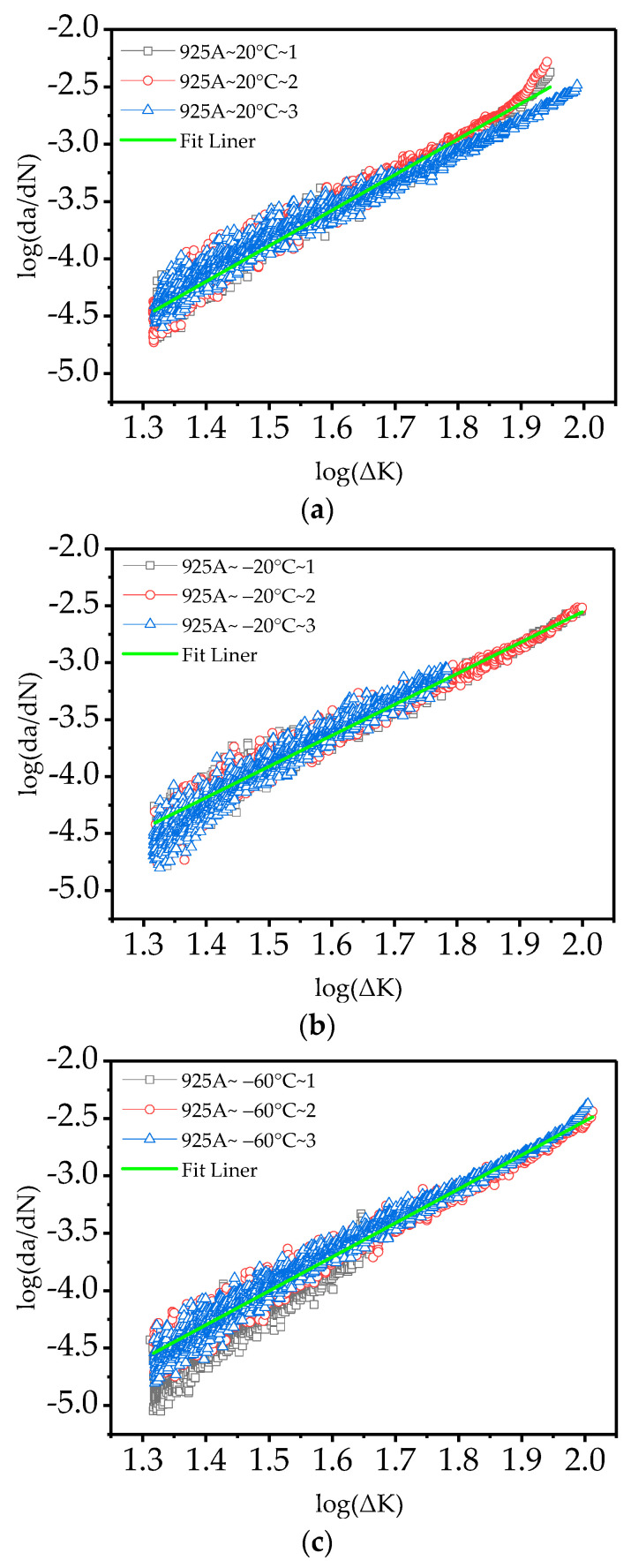
The curve of the 925A steel fatigue crack growth: (**a**) 20 °C; (**b**) −20 °C; (**c**) −60 °C.

Data processing is performed on nine test samples, with *m* and log*C* serving as fitting parameters. Linear fitting is performed on the test data points using the least squares method to obtain the fatigue crack propagation parameters of 925A steel at low temperatures. The fitting results are shown in Table 3 and Figure 4.

The values of the fitted correlation coefficient *R*^2^, except for one group at 0.94, reached or even exceeded 0.97. This finding indicates a high correlation between the fatigue crack propagation data obtained from the fatigue crack propagation test of 925A steel and the Paris law.

Life prediction for fatigue cracks was made easier and more quantitative with the introduction of the Paris law. The fatigue behavior of a material is usually represented with a diagram in which the rate of crack growth is plotted against the stress intensity factor range ∆*K* on a log-log graph. The typical graph shows three areas corresponding to the different crack propagation speeds: a regime with a slow crack growth rate (near threshold), a regime with mid growth rate (Paris regime), and a last part with a high growth rate [38,39,40]. The model in Figure 4 indicates that the entire fatigue propagation is in the region of moderate propagation rate. The data in the analysis chart also found that the crack growth rate curve shows good normalization and a good linear effect.

Figure 4 shows that under a stress ratio of 0.1, as the temperature decreases, the fatigue crack propagation rate of the 925A steel significantly decreases within the boundaries of a certain stress intensity factor. This finding suggests that the resistance of 925A steel to fatigue crack propagation in low-temperature environments is strengthened. At temperatures ranging from 20 °C to −60 °C, as the temperature decreases, the fracture toughness of the 925A steel increases, and the crack propagation rate decreases. However, at −60 °C, the fracture toughness somewhat decreases, and the crack propagation rate increases beyond a certain stress intensity factor compared to that at room temperature (20 °C). This result proves that 925A steel undergoes ductile fracture at −60 °C, but its overall life is greater than that at room temperature (20 °C), mainly due to the slow expansion of 925A steel under low-temperature conditions within the low-stress intensity factor range.

The Arctic temperature ranges from 20 °C to −60 °C [35]. Therefore, the temperature range used for this experiment ranges from room temperature to −60 °C. Within this range, it can be determined that the crack propagation rate of 925A steel is slow, thereby meeting the requirements for fatigue crack propagation. However, preventing low-temperature cold brittle fracture at extreme temperatures below −60 °C has become a key focus of low-temperature design.

## 4. Morphological Analyses of the Fracture

The fracture properties can be ascertained from a comprehensive evaluation analysis of the fracture morphology of the sample, including the suddenness of the fracture, the speed of crack propagation, and the characteristics of the fracture surface. In addition, by comparing and analyzing fracture specimens under different conditions, the fracture mechanism can be further revealed; specifically, the mechanisms by which fracture occurs and develops within the material can be determined.

**Figure 5:**
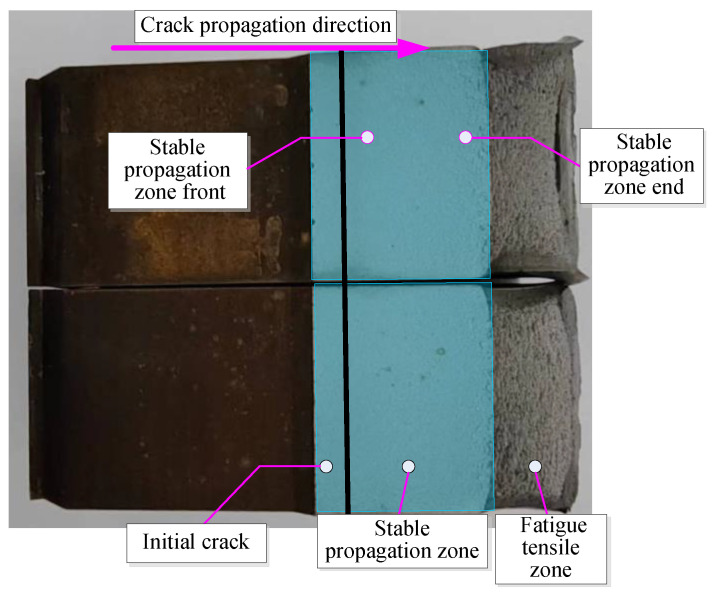
C(T) specimen fracture macro-morphology.

Figure 5 shows the macroscopic fracture morphologies of the fatigue cracks in specimen C(T) of the 925A steel. The stress intensity Factor *K* at the crack tip gradually increases along the crack propagation direction in the stable crack propagation zone. As the stress level increases, the cross-section gradually becomes rough and exhibits obvious brittle fracture characteristics. No obvious macroscopic defects can be observed from the macroscopic fracture surface, and the test material is relatively uniform.

**Figure 6:**
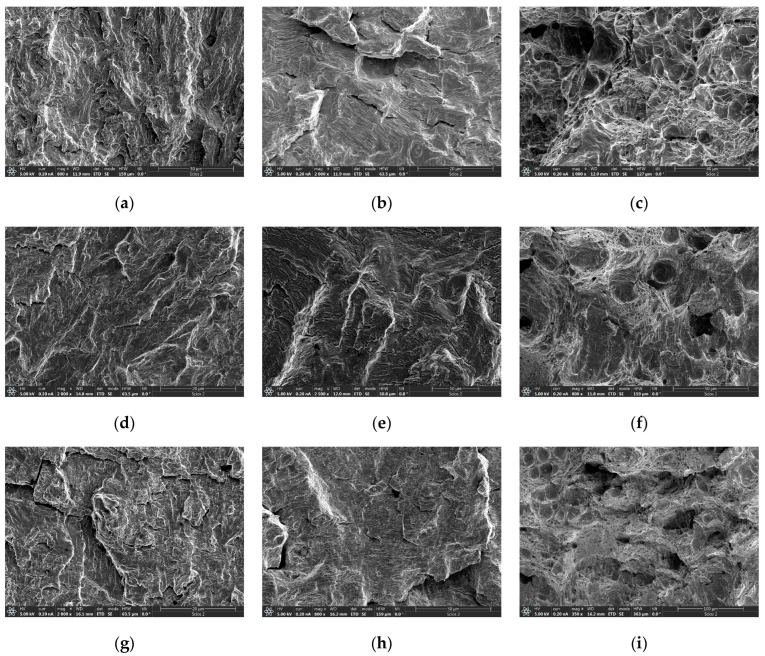
SEM images of the fracture morphologies of the C(T) samples. (**a**–**c**) Crack initiation zone: (**a**) 20 °C; (**b**) −20 °C; (**c**) −60 °C; (**d**–**f**) Stable crack propagation zone: (**d**) 20 °C; (**e**) −20 °C; (**f**) −60 °C; (**g**–**i**) Non-crack stable propagation zone: (**g**) 20 °C; (**h**) −20 °C; (**i**) −60 °C.

SEM is performed on the crack propagation zone at different temperatures. The microstructure of the crack initiation zone is shown in Figure 6a–c, the microstructure of the stable crack propagation zone is shown in Figure 6d–f, and the microstructure of the non-crack stable propagation zone is shown in Figure 6g–i. Clear fatigue striations perpendicular to the crack propagation direction can be observed that are accompanied by deconvolution steps and secondary regeneration cracks, which is a typical microstructure of deconvoluted fracture. There is no significant difference in the crack propagation fracture modes of the 925A steel C(T) specimens at three temperature points, namely, room temperature, −20 °C, and −60 °C, indicating that there is no occurrence of fatigue ductile–brittle transition within the test temperature range. At room temperature and −20 °C, the fracture surface exhibits quasi-cleavage fracture with short river patterns, small cleavage planes, and tearing edges. At −60 °C, the cross-sectional feature is the morphology of ductile dimples, with a relatively uniform distribution of ductile dimples, which may indicate that cracks propagate uniformly along weak areas inside the material. This finding indicates that the fatigue crack propagation fracture mode of the steel changes from quasi-cleavage to ductile fracture as the temperature increases from room temperature to −60 °C.

## 5. Conclusions

In this paper, a fatigue crack propagation rate test was carried out on 25-mm-thick 925A steel samples at room and low temperatures, and the following test conclusions were obtained by analyzing and processing the test data:

(1) The fatigue crack propagation rate of 925A steel was tested, and the *a–N* curve of 925A steel was obtained. The results showed that the service life of 925A steel increased with the decreasing temperature.
(2) At stress intensity factor amplitudes ranging from 20 to 100 MPa-m^1/2^, 925A steel exhibited excellent resistance to fatigue crack propagation.
(3) The macro fracture of the stable fatigue crack growth zone of the compact tensile specimen showed obvious cleavage fracture and morphological and ductile fracture characteristics. The measured crack growth rate was similar to that calculated by the Paris Law. The fitted Paris parameter could accurately describe the fatigue crack growth performance of 925A steel in this paper. The test data could provide a reference for the design of polar ships to resist low-temperature fatigue and cold brittle fracture.
(4) Within the polar normal temperature range, 925A steel met the requirements of a low-temperature fatigue crack growth rate in the polar region. However, at extreme polar temperatures below −60 °C, preventing brittle failure has become the focus of fatigue design, and relevant research should be carried out in the future.

## Figures and Tables

**Table 1:** Chemical composition of 925A steel (mass fraction, %).

C	Si	Mn	S	P	Cr	Ni	Mo	V	Cu
0.13~0.18	0.17~0.37	0.30~0.60	≤0.015	≤0.020	0.90~1.20	2.60~3.00	0.20~0.27	0.03~0.08	≤0.25

**Table 2:** Mechanical properties of 925A steel.

Temperature/T (°C)	Tensile Strength/σ_b_ (MPa)	Offset Yield Strength/σ_0.2_ (MPa)	Impact Energy/A_kv_ (J)	Elastic Modulus/E (GPa)	Poisson’s Ratio/*ν*
20	636	519	186	201	0.26
−20	633	515	178	194	0.27
−60	666	590	160	186	0.25

**Table 3:** Fitting parameters for the crack propagation characteristics of 925A steels at different temperatures.

Temperature/°C	Ratio	Frequency/Hz	log*C*	*m*	*R* ^2^
20	0.1	10	−8.55301	3.11012	0.97293
20	0.1	10	−8.36779	2.95344	0.96699
20	0.1	10	−8.54219	3.03567	0.97727
−20	0.1	10	−7.94087	2.68417	0.97673
−20	0.1	10	−7.97326	2.70931	0.97313
−20	0.1	10	−8.36779	2.95344	0.96699
−60	0.1	10	−9.85266	3.79749	0.94281
−60	0.1	10	−8.43396	2.95526	0.97634
−60	0.1	10	−8.54219	3.03567	0.97727

## Data Availability

Data are contained within the article.
